# A Study on the Medical Students' Perspectives of Their Educational Environment Using the Dundee Ready Educational Environment Measure (DREEM) at a Tertiary Care Teaching Hospital in Telangana, India

**DOI:** 10.7759/cureus.73272

**Published:** 2024-11-08

**Authors:** Sriguna Bannur, Shreya Veggalam, Sabitha Vadakedath, Venkataramana Kandi

**Affiliations:** 1 Medicine, Prathima Institute of Medical Sciences, Karimnagar, IND; 2 Biochemistry, Prathima Institute of Medical Sciences, Karimnagar, IND; 3 Clinical Microbiology, Prathima Institute of Medical Sciences, Karimnagar, IND

**Keywords:** bachelor of medicine and bachelor of surgery (mbbs), deficiencies, educational atmosphere, learning process, medical students' perspectives, privatization, quality of medical education, teaching quality

## Abstract

Introduction

India is currently experiencing a colossal increase in the number of medical colleges and a concurrent rise in the number of medical students pursuing Bachelor of Medicine and Bachelor of Surgery (MBBS). Shortages in the teaching faculty and infrastructural and logistic limitations like the availability of patients and clinical material are severely affecting the quality of medical education. Moreover, privatization has been instrumental in transforming medical education into a lucrative business compromising its quality. The present work intends to identify the deficiencies that could help the management create a better educational environment (EE) for the students. This study assessed the medical students' perspectives of the EE including the learning process, teaching quality, educational atmosphere, academic self-perception, and social self-perception.

Methods

This cross-sectional, questionnaire-based study included 212 medical students pursuing MBBS at the Prathima Institute of Medical Sciences (PIMS), Karimnagar, Telangana, India. The study used the standardized and globally validated Dundee Ready Educational Environment Measure (DREEM). Appropriate statistical methods were used to analyze and interpret the data.

Results

The cumulative DREEM score in this study was 112.64/200 (56.32%). The cumulative mean score of the responses was 2.39±0.42. The results demonstrated modest scores regarding the students’ academic self-perceptions (SASP; 20.42/36; 56.72%), the students' perception of teachers (SPT; 26.23/44; 59.61%), and the students' perception of learning (SPL; 27.65/48; 57.60%). The scores concerning the students’ perception of the atmosphere (SPA; 25.11/48; 52.31%), and the students' social self-perception (SSSP; 13.41/28; 47.89%) were also significantly inferior.

Conclusion

The study results revealed a satisfactory EE in the medical institution. Significant deficiencies concerning the college atmosphere and SSSP were observed. There is a scope for improvement in the domains of SASP, SPT, and SPL.

## Introduction

The educational environment (EE) is pivotal in sculpting the medical students' personalities, attitudes, and professional and social skills. It significantly influences academic performance, achievements, professional growth, mental and physical health, and human relationships. The quality of medical education (ME) depends on manifold factors that reflect the learning experience of budding doctors. Effective evaluation of the EE could reflect the functioning of medical institutions and highlight key areas where necessary interventions should be taken to improve the student experience [1-3].

Studies reveal that in ME, besides clinical training, students perform activities under supervision and obtain hands-on experience in the healthcare system and patient management. ME training intends to make students understand the nobility of the profession and shape them mentally and professionally [4]. Learning should be student-centered, enabling them to cultivate intrinsic self-motivation, learning by doing, meaningful engagement, multi-skill development, and better retention of knowledge [5]. Appropriate support systems, role models, and dedicated faculty members are required for students to develop the right skills, attitudes, and professional behavior that will allow future medical graduates to become patient-centered healthcare providers.

The Dundee Ready Educational Environment Measure (DREEM) is a tool designed to evaluate the EE in medical schools across the globe [6-8]. It is a globally recognized, accepted, and validated instrument to evaluate the EE in medical institutions providing undergraduate ME. The DREEM consists of five domains or subscales: students' perception of learning (SPL), students' perception of teachers (SPT), students' academic self-perceptions (SASP), students' social self-perception (SSSP), and students' perception of atmosphere (SPA). Results obtained from the DREEM assessment help the administration assess the status of the EE. It enables effective training of medical students, improves the standards, quality of teaching, and infrastructure at the institution, and comprehensively enhances the student's EE.

Gathering and analyzing the students' perspectives of the prevailing EE is crucial. This is the first study of its kind from Telangana. The state, like the rest of the country, has seen an enormous increase in the number of medical colleges over the past decade. This study assessed the students' perceptions of the EE in a tertiary care teaching hospital.

## Materials and methods

This cross-sectional, questionnaire-based study utilized the DREEM questionnaire. It was undertaken at the Prathima Institute of Medical Sciences (PIMS), Telangana, India in March 2024. The study included 212 medical students pursuing Bachelor of Medicine and Bachelor of Surgery (MBBS) and belonging to different phases of the course (MBBS first, second, and third year part I, third year part II or final year, and interns). The institutional ethics committee of PIMS approved the study (IEC/PIMS/2021-007-01112021e).

Inclusion and exclusion criteria

The study included students pursuing MBBS at PIMS and those who volunteered and consented to participate. It excluded students from paramedical courses, graduates, doctors, and postgraduate students.

DREEM

The DREEM is a 50-item questionnaire that is globally validated, accepted, reliable, and culturally-free inventory developed by an international Delphi approach published in 1997. The Dundee in DREEM represents its origin from the University of Dundee, United Kingdom [6,7].

It is a tool that measures the EE of medical institutions. The 50-item questionnaire has a maximum score of 200, which means the institution has the best EE according to the students. The scores were calculated based on the participants' responses [7,9].

The DREEM includes five domains/subscales: a 12-item SPL and SPA with a maximum score of 48 each, an 11-item SPT with a maximum score of 44, an eight-item SASP with a maximum score of 32, and a seven-item SSSP with a maximum score of 28.

Google Forms were used to collect the data including basic demographic details like age, gender, year of admission, eligibility for scholarships, and parents' annual income along with the DREEM questionnaire. The questionnaire was circulated to the student groups of all five years (2018-2023) studying MBBS and those doing internships through WhatsApp (WhatsApp Inc., Menlo Park, California, USA).

Responses to each of the 50 items were based on a five-point Likert scale (strongly agree-four points, agree-three points, uncertain/not sure-two points, disagree-one point, strongly disagree-zero points). In the case of negatively-framed questions (question numbers 4, 8, 9, 17, 25, 35, 39, 48, 50), the points were reversely awarded for the same set of options (strongly agree-zero points, agree-one point, uncertain/not sure-two points, disagree-three points, strongly disagree-four points) [6,7].

Interpretation of the DREEM scores

If the mean score for each item was ≥3, it was considered a positive perception, a score of ≥2.0 but <3.0 was considered an area requiring improvement, and a mean score of <2.0 was considered very poor. The interpretation of the DREEM scores was carried out as per the existing recommendations with some modifications (Table 1) [6-9].

**Table 1:** Interpretation of the DREEM scores DREEM: Dundee Ready Educational Environment Measure; SPL: students’ perception of learning; SPT: students’ perception of teachers; SASP: students’ academic self-perceptions; SPA: students’ perception of atmosphere; SSSP: students’ social self-perception.

DREEM domain/subscale	Maximum score	Range	Interpretation
Total score	200	0-50	Very poor
		51-100	Less than satisfactory
		101-150	Satisfactory
		151-200	Excellent
SPL	48	0-12	Very poor
		13-24	Less than satisfactory
		25-36	Satisfactory
		37-48	Excellent
SPT	44	0-11	Very poor
		12-22	Less than satisfactory
		23-33	Satisfactory
		34-44	Excellent
SASP	32	0-08	Very poor
		09-16	Less than satisfactory
		17-24	Satisfactory
		25-32	Excellent
SPA	48	0-12	Very poor
		13-24	Less than satisfactory
		25-36	Satisfactory
		37-48	Excellent
SSSP	28	0-07	Very poor
		08-14	Less than satisfactory
		15-21	Satisfactory
		22-28	Excellent

Statistical analysis

The data received was entered manually in a Microsoft Office 2019 Excel sheet (Microsoft Corp., Redmond, WA, USA), and statistical inferences were drawn using IBM SPSS Statistics for Windows, Version 20 (Released 2011; IBM Corp., Armonk, New York, United States). The individual question cumulative scores, domain scores, total cumulative DREEM scores, and domain-wise scores with standard deviations were calculated using descriptive statistical methods like mean, maximum value, minimum value, and percentages. The probability (p) value was used for statistical significance (p<0.05).

## Results

Out of 212 MBBS students who participated in the study, 66 students were from the first year (31.1%), 103 from the second year (48.6%), 19 from the third year part I (9.0%), 14 from the third year part II (6.6%) and 10 were interns (4.7%). In the study population, 63 (29.7%) were male students and 149 (70.3%) were female students.

**Figure 1:**
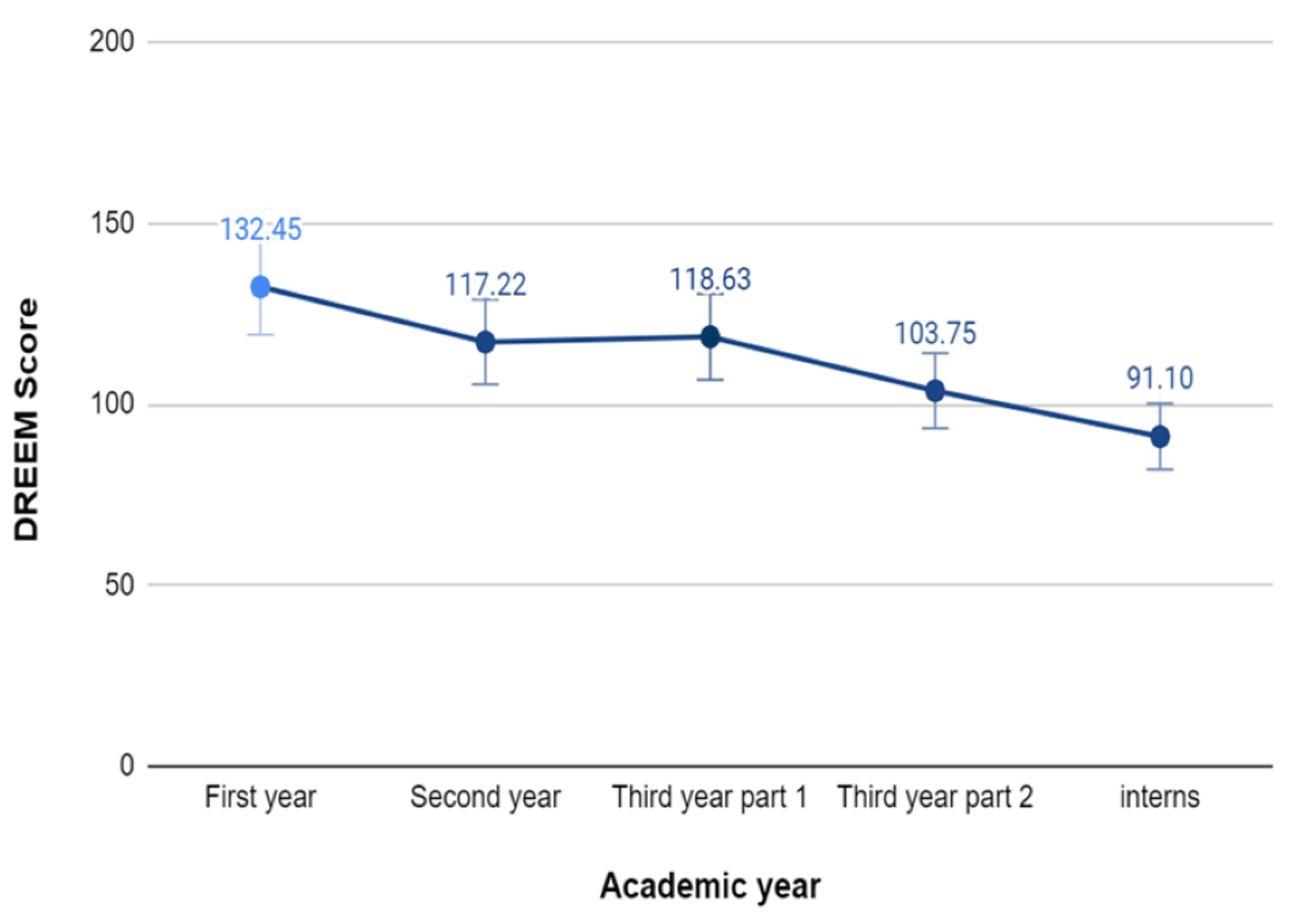
Graph showing the DREEM scores based on the stage of the MBBS course DREEM: Dundee Ready Educational Environment Measure; MBBS: Bachelor of Medicine and Bachelor of Surgery

Students' perception of the EE declined over the years, which implies growing dissatisfaction as they progress through their academic journey. Interns, in particular, were the most dissatisfied and conveyed that the college has several problems. The DREEM scores obtained from the MBBS students of the first year, second year, third year part I and part II, and the interns were 132.45±17.99, 117.22±23.25, 118.63±20.36, 103.75±30.13, and 91.1±29.44, respectively (Figure 1).

The total cumulative DREEM score of the institute was 112.64±24.48 out of 200 (56.32%) with the scores ranging from 32 to 177. This result represents a satisfactory EE according to the interpretation of the DREEM scores. The cumulative score of the responses was 2.40±0.49. The ages of the students who participated in the study ranged from 17 to 26 years with a mean age of 19.94±1.55 years.

The individual domain scores for SPL (27.65/48; 57.60%), SPT (26.23/44; 59.61%), SASP (20.42/36; 56.72%), SPA (25.11/48; 52.31%), and SSSP (13.41/28; 47.89%) are detailed in Table 2.

**Table 2:** Domain-based cumulative DREEM scores of the study respondents ^#^p-value obtained by comparing the responses from the MBBS first year students with that of the interns. MBBS: Bachelor of Medicine and Bachelor of Surgery; DREEM: Dundee Ready Educational Environment Measure; SPL: students’ perception of learning; SPT: students’ perception of teachers; SASP: students’ academic self-perceptions; SPA: students’ perception of the atmosphere; SSSP: students’ social self-perception.

Domain	MBBS first year (n=66) (mean±SD)	MBBS second year (n=103) (mean±SD)	MBBS third year part I (n=19) (mean±SD)	MBBS third year part II (n= 14) (mean±SD)	Interns (n=10) (mean±SD)	Mean scores of each domain (mean±SD)	p-value^#^
SPL	32.36±3.81	28.18±5.26	29.05±5.52	26.34±6.39	22.30±8.91	27.65±5.98	<0.0001
SPT	30.79±4.41	27.98±5.29	26.68±5.67	23.92±4.39	21.80±6.31	26.23±5.21	<0.0001
SASP	22.67±3.24	20.93±4.36	22.63±3.91	18.67±6.25	17.20±5.29	20.42±4.61	<0.0001
SPA	30.10±5.55	26.30±7.44	26.00±6.30	21.25±7.05	21.90±7.57	25.11±6.78	0.0001
SSSP	16.55±4.76	13.83±5.18	14.26±3.82	11.50±6.91	10.90±3.27	13.41±4.79	0.0005
Total DREEM score for each year	132.45±17.99	117.22±23.25	118.63±20.36	103.75±30.13	91.10±29.44	112.64±24.23	<0.0001

Influence of other parameters on the DREEM score

The influence of other parameters such as the year of study, age, gender, income of parents, and scholarship status of the students on the DREEM score was also evaluated. A significant decrease (p<0.0001) in the DREEM score was observed as the year of study advanced. This was evident from the high scores of students in their first year (132.45±17.90) versus those doing their internship (91.10±29.44). Students above 20 years of age scored lower (115.26±23.97) than those in their teens (17-19 years; 110.02±24.59); though the difference was not significant (p=0.12). The difference in the DREEM score between the male (110.69±24.76) and the female students (112.70±24.34) was also insignificant (p=0.58).

There was no statistically significant difference (p=0.50) in the students' perception of the EE based on their annual parental income; more than five lakhs/year (112.02±27.38) versus less than five lakhs/year (115.04±22.22). Students who received scholarships had a slightly better perception of the EE (118.58±20.40) than those who did not receive financial aid (111.27±24.87); though the difference was again not statistically significant (p=0.17).

The domain-wise scores of the study participants are shown in Tables 3-7.

**Table 3:** Responses to students’ perception of learning domain ^n^: negatively-framed questions, ^*^: mean scores <2.00 MBBS: Bachelor of Medicine and Bachelor of Surgery; DREEM: Dundee Ready Educational Environment Measure; SPL: students’ perception of learning

Domain/subscale	DREEM questions	MBBS first year (n=66)	MBBS second year (n=103)	MBBS third year part I (n=19)	MBBS third year part II (n= 14)	Interns (n=10)	Average of responses (mean±SD)
SPL	1. I am encouraged to participate in class	3.06	2.45	2.42	2.50	1.90	2.47±0.41
	7. The teaching is often stimulating	2.73	2.26	2.00	2.25	2.00	2.25±0.30
	13. the teaching is student-centered	2.91	2.36	2.47	1.92	1.70	2.27±0.48
	16. The teaching helps to develop my competence	2.85	2.55	2.68	2.17	1.60	2.37±0.50
	20. The teaching is well focused	2.95	2.38	2.68	2.34	1.20	2.31±0.67
	21. I am being well prepared to be a good/competent physician	3.17	2.74	2.74	2.42	1.40	2.50±0.67
	24. The teaching time is utilized properly	2.68	2.54	2.84	2.25	1.30	2.32±0.61
	25. The teaching emphasizes on factual learning^n ^	1.02	1.19	1.11	1.83	2.10	1.45±0.48^*^
	38. I am clear about the learning objectives of the courses/subjects	2.86	2.71	2.89	2.25	1.70	2.48±0.51
	44. The teaching encourages me to be an active learner	2.86	2.50	2.58	2.17	1.70	2.36±0.44
	47. Long-term learning is emphasized over short-term learning	3.00	2.65	2.89	2.33	1.70	2.52±0.52
	48. The teaching is too teacher-centered^n^	2.27	1.85	1.74	1.92	1.20	1.80±0.39^*^

**Table 4:** Responses to students’ perception of teachers domain ^n^: negatively-framed questions, ^*^: mean scores <2.00, ^#^: mean scores >3.00 MBBS: Bachelor of Medicine and Bachelor of Surgery; DREEM: Dundee Ready Educational Environment Measure; SPT: students’ perception of teachers

Domain/subscale	DREEM questions	MBBS first year (n=66)	MBBS second year (n=103)	MBBS third year part I (n=19)	MBBS third year part II (n= 14)	Interns (n=10)	Average of responses (mean±SD)
SPT	2. The teachers are knowledgeable	3.29	3.10	2.89	2.42	2.80	3.08±0.28^#^
	6. The teachers are patient with patients	2.92	2.97	2.68	2.67	2.60	2.77±0.17
	8. The teachers demotivate the students^n^	2.79	2.86	2.37	1.92	2.30	2.45±0.39
	9. The teachers are dictatorial^n^	2.05	2.13	1.79	1.08	1.30	1.67±0.46^*^
	18. The teachers have good communication skills with patients	2.94	2.93	2.95	2.83	2.40	2.81±0.23
	29. The teachers are good at providing feedback to students	2.42	2.34	2.21	1.75	1.50	2.04±0.40
	32. The teachers provide constructive criticism	2.12	1.96	2.53	2.42	1.70	2.15±0.34
	37. The teachers give clear examples	2.70	2.63	2.74	2.25	2.30	2.52±0.23
	39. The teachers get angry during lectures^n^	1.87	1.76	1.37	1.58	1.80	1.68±0.20^*^
	40. The teachers are well prepared for their lectures	2.84	2.85	2.63	2.67	1.50	2.50±0.57
	49. I feel I am able to ask the questions I want	2.48	2.45	2.53	2.33	1.60	2.28±0.39

**Table 5:** Responses to students’ academic self-perceptions domain ^#^: mean scores >3.00 MBBS: Bachelor of Medicine and Bachelor of Surgery; DREEM: Dundee Ready Educational Environment Measure; SASP: students' academic self-perceptions

Domain/ subscale	DREEM questions	MBBS first year (n=66)	MBBS second year (n=103)	MBBS third year part I (n=19)	MBBS third year part II (n= 14)	Interns (n=10)	Average of responses (mean±SD)
SASP	5. Learning strategies which worked for me before continue to work even now	2.15	2.19	2.63	2.17	2.10	2.25±0.22
	10. I am confident about my passing this year	3.29	3.12	3.05	2.83	3.20	3.10±0.17^#^
	22. The teaching helps develop my confidence	3.00	2.38	2.53	1.83	2.00	2.35±0.46
	26. Last year’s learning has been a good preparation for this year’s progress	2.67	2.80	3.11	2.17	1.90	2.52±0.49
	27. I am able to memorize basics of the subjects	2.62	2.47	2.63	2.50	2.20	2.48±0.17
	31. I have learnt a lot about empathy in my profession	3.07	2.67	3.00	2.42	2.10	2.65±0.40
	41. My problem solving skills are being well developed	2.74	2.42	2.79	2.08	1.40	2.29±0.57
	45. Much of what I have to learn seems relevant to a career in healthcare	3.15	2.89	2.89	2.67	2.30	2.78±0.32

**Table 6:** Responses to students’ perception of the atmosphere domain ^n^: negatively-framed questions, ^*^: mean scores <2.00 MBBS: Bachelor of Medicine and Bachelor of Surgery; DREEM: Dundee Ready Educational Environment Measure; SPA: students' perception of the atmosphere

Domain/ subscale	DREEM questions	MBBS first year (n=66)	MBBS second year (n=103)	MBBS third year part I (n=19)	MBBS third year part II (n= 14)	Interns (n=10)	Average of responses (mean±SD)
SPA	11. The atmosphere is relaxed during the clinical teaching	2.64	2.44	2.26	2.08	2.10	2.30±0.24
	12. The college follows a timetable	3.21	2.85	2.89	1.58	1.90	2.50±0.70
	17. Cheating/malpractice is a problem in the college^n^	2.18	2.00	1.74	1.83	1.60	1.87±0.23^*^
	23. The atmosphere is relaxed during lectures	2.68	2.37	2.05	2.42	2.70	2.44±0.27
	30. There are opportunities for me to develop interpersonal skills	2.79	2.23	2.37	1.58	1.90	2.17±0.46
	33. I feel comfortable in teaching sessions socially	2.73	2.45	2.63	2.67	2.00	2.49±0.30
	34. The atmosphere is relaxed during seminars/ tutorials	2.58	2.55	2.53	2.58	2.00	2.45±0.25
	35. I find my learning experience disappointing^n^	2.67	2.16	1.84	1.58	1.30	1.91±0.53^*^
	36. I am able to concentrate well	2.39	2.14	2.37	2.00	2.20	2.22±0.16
	42. The enjoyment outweighs the stress of the courses	2.10	1.95	1.89	1.67	1.30	1.78±0.31^*^
	43. The atmosphere motivates me as a learner	2.73	2.13	2.63	1.92	1.30	2.14±0.58
	50. Some students irritate the teachers^n^	1.42	1.04	0.79	1.42	1.40	1.21±0.29^*^

**Table 7:** Responses to the students’ social self-perception domain ^*^: mean scores <2.00 MBBS: Bachelor of Medicine and Bachelor of Surgery; DREEM: Dundee Ready Educational Environment Measure; SSSP: students' social self-perception

Domain/subscale	DREEM questions	MBBS first year (n=66)	MBBS second year (n=103)	MBBS third year part I (n=19)	MBBS third year part II (n= 14)	Interns (n=10)	Average of responses (mean±SD)
SSSP	3. There is a good support system for students who get stressed	2.45	1.76	0.89	1.25	0.90	1.45±0.66^*^
	4. I am too tired to enjoy the courses	2.11	1.93	1.89	2.00	1.10	1.81±0.40^*^
	14. I am rarely bored during learning	2.03	1.48	1.74	1.25	0.90	1.48±0.44^*^
	15. I have good friends in this college	2.86	2.58	3.26	1.75	3.00	2.68±0.58
	19. My social life is good	2.47	2.24	2.63	2.00	2.10	2.29±0.26
	28. I rarely feel lonely	2.23	1.87	2.00	1.25	1.90	1.85±0.36^*^
	46. My accommodation at the college is pleasant	2.39	1.96	1.89	2.00	1.00	1.85±0.51^*^

## Discussion

The results showed notable deficiencies concerning SPA (25.11/48; 52.31%) and SSSP (13.41/28; 47.89%). Further, the results indicated scope for large-scale improvements in SASP (20.42/36; 56.72%), SPT (26.23/44; 59.61%), and SPL (27.65/48; 57.60%).

The physical learning environment plays a significant role in molding students to achieve better academic outcomes. Factors influencing student learning experiences include psychological and social characteristics intrinsic to the EE. Therefore, understanding the students' subjective (individual) and objective (comprehensive) perspectives concerning different aspects of the EE is an essential area of educational research requiring extensive investigation [10]. Active learning environments with enhanced student involvement influence subject comprehension [11]. 

The results of the SPL domain in this study indicate that, in all the academic years, the teachers emphasized factual rather than concept-based teaching. This includes critical thinking and speculation, which are important for students to understand to apply those points and knowledge in real life. Additionally, discordant opinions were noted with some students believing that the teaching was teacher-centered (1.80±0.39) while others thought the teaching was student-centered (2.27±0.48).

Students in different phases of the MBBS course did not feel well-prepared to be competent physicians. This perception was strongest among interns compared to those studying in the first year. This perception may have been influenced by experiences like disruptive and demeaning behavior by colleagues and peers [12].

An analysis of the SPT in this study indicates most students agreed that the teachers were knowledgeable. However, they considered the teachers authoritative and unnecessarily furious during the lectures. The teachers' mindsets and attitudes greatly influence students' learning experience [13]. 

Regarding SASP, the results demonstrated that the students were self-motivated and confident about passing the degree with honors. This confirms that the students admitted to MBBS are sure about finishing their degrees.

The SPA scores (1.87±0.23) reflect the students' concern about the malpractices persisting in the institution. This could have led to their disappointing learning experience. The students agreed that enjoyment outweighed the stress of the course. This indicates that despite the course being stressful, the enjoyment/fulfillment of the students was strong enough to compensate for it. Most respondents agreed that few students in every class trouble the teachers, affecting their learning experience. The optimistic perception of the college atmosphere diminished through the years of professional education. 

In the case of the SSSP domain, the significantly low scores (mean scores<2.0) indicate the institution's failure to provide a support system for students who are under stress. This includes providing qualified counselors and stress management sessions/workshops to reduce or control stress. Stress severely affects the mental health of students, especially those pursuing professional courses like MBBS [14]. The students felt that fatigue and exhaustion from this course were so overwhelming that they could not find any pleasure in their studies. They often felt lonely (1.85±0.36) despite having friends in the college (2.68±0.58) and having a reasonably good social life (2.29±0.26). They acknowledged living conditions was not satisfactory at their college accommodation (1.85±0.51).

The lower DREEM scores among interns (91.1±29.44) compared to the first-year MBBS students (132.45±17.99) may be attributed to the increased workload and academic pressure, lack of support system and resources, physical and mental fatigue, and bureaucratic pressures and inefficiencies. Interns face the most turbulent work schedules and responsibilities [12]. Their views precisely highlight the institution’s shortcomings and contribute to a more critical view of the EE. In contrast, first-year students had the most positive perception, which can be due to their cheerful enthusiastic mindsets, supportive peers, strong support systems like mentors and counselors, lack of awareness of institutional challenges, optimism and hopefulness about their future careers, and easier grasp of broad subjects.

The DREEM scores did not exceed 150 (indicating excellent EE) even in institutions located in the most developed and high-income countries. An analysis of the DREEM scores of institutions located worldwide confirms that income level alone does not determine the quality of the EE as relatively low and high scores were observed from Japan and Sudan, respectively. Even with similar income levels, we see a significant variability in the DREEM scores [15-21]. The institutions with lower DREEM scores must take steps to enhance their EEs (Table 8).

**Table 8:** DREEM scores based on the income level (as per World Bank country classification) DREEM: Dundee Ready Educational Environment Measure; ICSEI: International Congress for School Effectiveness and Improvement

Income level classification	Country	Year	Institution/University	DREEM score (maximum 200)	Reference
High income	Japan	2019	Kindai University	113.40	Ikeda et al. [15]
	Australia	2011	Monash University	137.30	Brown et al. [16]
Upper-middle income	Colombia	2020	ICSEI University	125.00	Arenas et al. [17]
	Iraq	2020	Hawler College of Medicine	101.40	Saleh et al. [18]
Lower-middle income	India	2024	Prathima Institute of Medical Sciences	112.64	This study
	Nigeria	2023	Unnamed institution	116.20	Oguntoye et al. [19]
Low income	Syria	2023	University of Aleppo	101.30	Alfakhry et al. [20]
	Sudan	2018	University of Bahri	125.30	Salih et al. [21]

An analysis of the DREEM scores in different regions of India revealed variable scores for institutions in Delhi (130.34) [22], West Bengal (123.10) [23], Gujarat (124.58) [24], and Tripura (119.58) [25]. The scores observed in these institutions revealed satisfactory EE. Delhi scored the highest since it is the capital city with higher economic and educational development. Further, it is also home to some of India's top-tier medical colleges equipped with sophisticated infrastructure and reputed faculty.

In Telangana, less urbanized areas face significant challenges like infrastructure and faculty shortage. While the state has well-regarded medical colleges, many institutions still struggle with limited resources. The white-coat movement could have contributed to the lower DREEM score obtained in this study. This movement led to the rapid establishment of medical colleges, especially in the rural areas. This resulted in inferior quality of resources, infrastructure, and faculty deficiencies [26]. Between 2021 and 2024, the number of medical colleges doubled in Telangana (from 35 to 70), marking a 100% growth [27]. However, this rapid expansion has raised concerns about the sustainability and quality of the ME.

There are 706 medical colleges in India, producing around 110,000 medical graduates per year [28]. The disturbing fact is that only 50% of the colleges have sufficient faculty [29]. Ideally the student-to-teacher ratio has to be 15:1 to 20:1 [30]. An optimal student-to-teacher ratio helps ensure a good reception of the EE.

Study limitations

Since this study comprised self-reported data, students may have underestimated or overestimated themselves creating bias in the results. The DREEM questionnaire with 50 items might be tiring for students to fill, thus affecting their responses. The results obtained from this study cannot be generalized to the EE of statewide and nationwide medical colleges. There is a possibility of response bias associated with honesty and fear of peers, teachers, and the college administration. This is a closed-ended questionnaire-based study; therefore, the participants' views may not be definitive.

## Conclusions

The results of this study revealed a satisfactory EE in the institution. Gender, economic status, and scholarship did not affect the students' perception of the EE. Significant deficiencies concerning the college atmosphere and SSSP were observed. There is a scope for improvement in the SASP, SPT, and SPL domains. This paper stands as evidence for policymakers to fulfill the essential needs of medical students. This study recommends that education stakeholders must enhance the quality of the EE. Our study emphasizes that the quality of the ME and the proficiency of doctors should be given priority over quantity. Through the DREEM analysis, medical college administrators can ascertain deficiencies in the institution and fix them to create a favorable EE for students and enable them to become better doctors. 

Based on the results obtained in this study, several targeted measures can be implemented, such as student support services to address their emotional and psychological needs, improving the infrastructure of classrooms and accommodations, organizing extracurricular activities outside of academics, and arranging workshops on critical thinking and skill development to make students more competent. Implementing these can foster a more supportive, engaging, and productive learning environment for medical students. A periodic assessment of the EE through DREEM is recommended to assess the deficiencies in a medical institution.

## Figures and Tables

**Figure 2:**
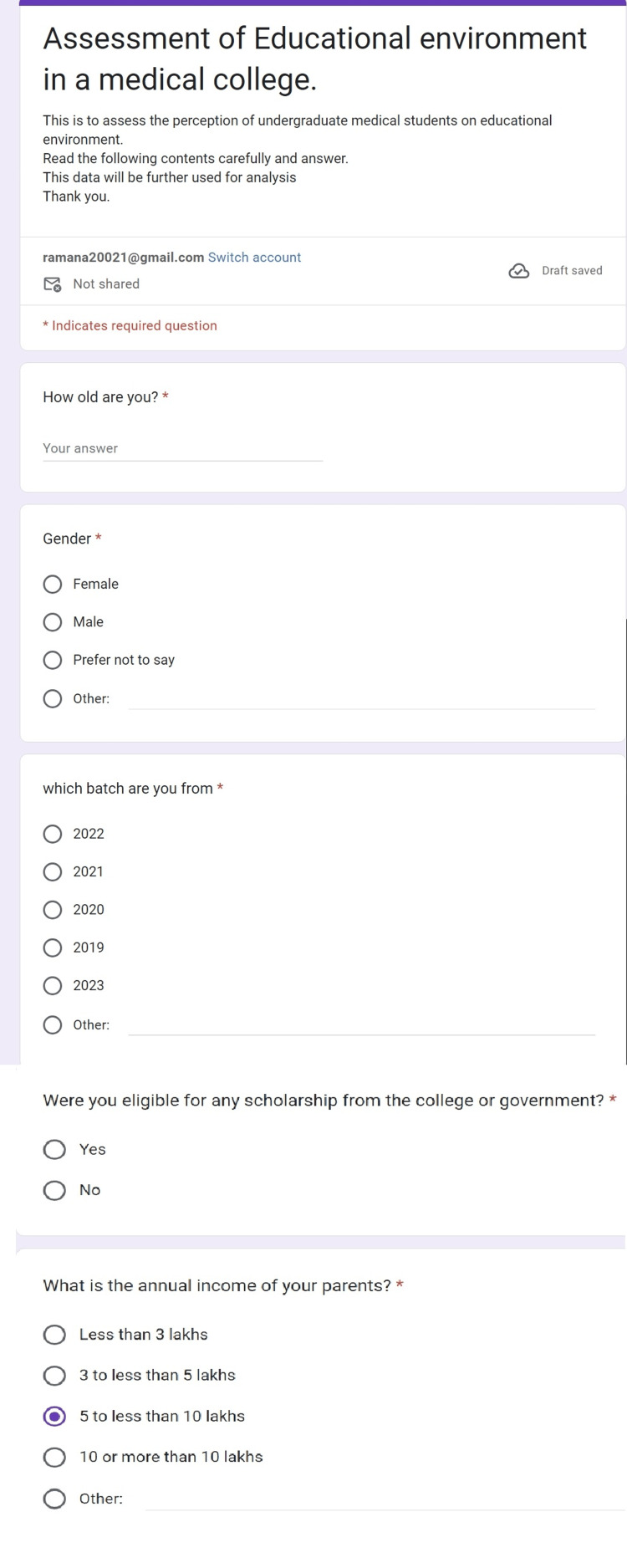
Google Form used to collect basic demographic and financial details from study participants
